# Tensile Strength and Mode I Fracture Toughness of Polymer Concretes Enhanced with Glass Fibers and Metal Chips

**DOI:** 10.3390/ma17092094

**Published:** 2024-04-29

**Authors:** Mazaher Salamat-Talab, Ali Zeinolabedin-Beygi, Faraz Soltani, Alireza Akhavan-Safar, Ricardo J. C. Carbas, Lucas F. M. da Silva

**Affiliations:** 1 Department of Mechanical Engineering, Arak University of Technology, Arak 3818146763, Iran; m.salamattalab@gmail.com; 2 Department of Mechanical Engineering, Tarbiat Modares University, Tehran 1411713116, Iran; alizeynolabedin8@gmail.com; 3 Department of Mining Engineering, Arak University of Technology, Arak 3818146763, Iran; faraz.soltani@arakut.ac.ir; 4 Institute of Science and Innovation in Mechanical and Industrial Engineering (INEGI), 4200-465 Porto, Portugal; 5 Department of Mechanical Engineering, Faculty of Engineering, University of Porto, 4200-465 Porto, Portugal; lucas@fe.up.pt

**Keywords:** polymer concrete, glass fibers, metal chips, semi-circular bend, fracture energy, tensile strength

## Abstract

This study experimentally investigates the influence of metal chips and glass fibers on the mode I fracture toughness, energy absorption, and tensile strength of polymer concretes (PCs) manufactured by waste aggregates. A substantial portion of the materials employed in manufacturing and enhancing the tested polymer concrete are sourced from waste material. To achieve this, semi-circular bend (SCB) samples were fabricated, both with and without a central crack, to analyze the strength and fracture behavior of the composite specimens. The specimens incorporated varying weight percentages comprising 50 wt% coarse mineral aggregate, 25 wt% fine mineral aggregate, and 25 wt% epoxy resin. Metal chips and glass fibers were introduced at 2, 4, and 8 wt% of the PC material to enhance its mechanical response. Subsequently, the specimens underwent 3-point bending tests to obtain tensile strength, mode I fracture toughness, and energy absorption up to failure. The findings revealed that adding 4% brass chips along with 4% glass fibers significantly enhanced energy absorption (by a factor of 3.8). However, using 4% glass fibers alone improved it even more (by a factor of 10.5). According to the results, glass fibers have a greater impact than brass chips. Introducing 8% glass fibers enhanced the fracture energy by 92%. However, in unfilled samples, aggregate fracture and separation hindered crack propagation, and filled samples presented added barriers, resulting in multiple-site cracking.

## 1. Introduction

Different types of concrete materials, which are usually a mixture of adhesives, aggregates, and sometimes reinforcements, are used in the construction of structures. The advantages of concrete materials include ease of use, low cost, and high compressive strength. However, they have disadvantages such as high porosity, poor tensile strength, chemical corrosion, and high sensitivity to environmental conditions [1]. Various authors have explored composite concretes [2,3]. Nevertheless, within the realm of composite concretes, those composed of materials, specifically polymer concretes, have garnered comparatively limited research attention. Polymer concrete (PC) is produced from a mixture of mineral aggregates with polymer adhesives, which is relatively newer compared to conventional cement concrete materials [4,5]. Additives, polymers and aggregates are used in making polymer concrete materials [6]. Generally, large and small aggregates constitute more than 75–80% of PC materials. Epoxy and polyester resins can be referred to as common matrix in polymer concrete materials. In addition, different types of fillers are added to the matrix and aggregates to improve the mechanical properties and performance of these materials [7,8]. The advantages of PC materials include high corrosion resistance, good resistance to chemical reactivity, preservation of concrete against carbonation phenomena, and ease of use in thin sections [9,10]. Although PC materials are more expensive than common concrete, their use is useful for stabilizing existing structures. Experimental studies have shown that the fracture energy and tensile strength of PC materials is 3.5–4.5 times that of conventional cement concrete structures, so they can be considered as a suitable alternative to cement concrete [11,12].

In general, three methods of changing the portion of concrete elements, changing the type of resin, and adding fillers are used to increase the mechanical performance of PCs. Considering the expensiveness of resins that have high mechanical properties, changing the type of resin is not a cost-effective method. Regarding the method of changing the percentage of concrete elements, the reduction of resin leads to concrete drying and the increase of resin also reduces some mechanical properties. Several studies have been conducted to achieve the optimal combination of PC materials to achieve good mechanical properties [13,14]. One simple method to improve the mechanical behavior of PC materials is to add filler, with the cost of this method depending on the type of filler. For example, waste material has a much lower price compared to nanoparticles. Many researchers have used waste material to increase mechanical properties [15,16,17,18,19,20,21]. Therefore, to increase the mechanical properties of polymer concrete materials, various waste materials have been used [22,23]. Son and Yeon [24] investigated the effect of methacrylic acid filler on the mechanical behavior of polymer material at low temperatures. The results showed that the addition of methacrylic acid increases Poisson’s ratio, tensile strength, and Young’s modulus of PC. D’Alessandro et al. [25] calculated the thermal conductivity and dynamic stiffness of concrete containing polymers made by electric wire sheets. They found that improved PC can be used for acoustic and thermal coatings of lightweight insulation. Kou and Poon [26] observed that adding fly ash as a filler to the polymer mixture improves chemical resistance with greater flexibility. The effect of burning wood ash as a filler on the mechanical performance of PCs was investigated by Teixeira et al. [27]. They found that the addition of wood ash increases the durability, while it has a detrimental effect on the mechanical properties. The effect of incorporating electronic plastic waste on the mechanical behavior of PC was investigated by Bulut and Şahin [28]. The results showed that by adding filler, compressive strength and bending strength decrease, but ductility increases. Heidari-Rarani et al. [29] investigated the mode I fracture toughness and tensile strength of an optimized epoxy PC using three different freeze/thaw cycles. For this purpose, they used single edge notch bending (SENB) and Brazilian disk (BD) samples. They found that the mode I fracture toughness and tensile strength of PC materials increase significantly with the heat-to-cool thermal cycle. In addition, it was found that an increase in the average temperature of the thermal cycles leads to a decrease in the mode I fracture toughness and tensile strength. Aliha et al. [30] investigated the effect of large silica aggregate mix-design made of epoxy resin, E-glass fibers, and mall sand filler on mode I cracking behavior using semi-circular bend (SCB) samples. The results showed that the mechanical performance of PC mix is not always improved by adding fiber to particular mix-designs. In addition, it was shown that the fracture energy and mode I fracture toughness in some cases of PC mix-design (PC with 2% short glass fibers) increased up to 53 and 41%. Asdollah-Tabar et al. [31] investigated the fracture energy and mode I fracture toughness of the PC material by adding recycled polyethylene terephthalate (PET) bottles. For this purpose, two different sizes were used. They found that the fracture toughness of the PC material increased by approximately 16% with the addition of coarse PET filler. Also, if a large PET filler is used, the mode I fracture toughness can be significantly increased compared to the addition of a small PET filler. 

As mentioned, many of the studies focused on improvement mechanical performance of PCs and no comprehensive study was made on the effect of brass metal chips and mixture of metal chips with glass fibers on the tensile strength and mode I fracture toughness of PCs. Therefore, this study aims to manufacture PC materials using waste mineral aggregates and two types of fillers, and to investigate their tensile strength and mode I fracture behavior experimentally. For this purpose, glass fibers and brass chips, which are the rounding out of the machining process, were used in different weight percentages (2 wt%, 4 wt%, and 8 wt%). Fracture and 3-point bending (3PB) tests were then performed with and without central crack SCB samples to obtain the mode I fracture toughness, tensile strength, and energy absorbed until failure.

## 2. Experimental Procedure

### 2.1. Sample Configuration and Material

Generally, epoxy and polyester resins are used to make PC, and epoxy resins are more common in the industry due to easier control of gel time and better material properties than polyester resin [32]. For this purpose, EPL 1012 epoxy resin was used together with EPH 112 hardener, with a weight ratio of 100:12 [33,34]. Table 1 shows the mechanical properties of EPL 1012 resin and EPH 112 hardener. The weight percentage of the resin used was 25 wt%. To complete the curing and obtain maximum strength, this resin was post-cured at room temperature for 7 days. The polyester resin used in this study was obtained from the Boytec company (Istanbul, Turkey) and was polymerized by adding 0.6 wt% hardener (methyl ethyl ketone peroxide). The resin was cured at room temperature for 40 h and, finally, according to the producer’s instructions, the sample was placed in the oven for 8 h at a temperature of 80 °C for post-curing [35].

**Figure 1:**
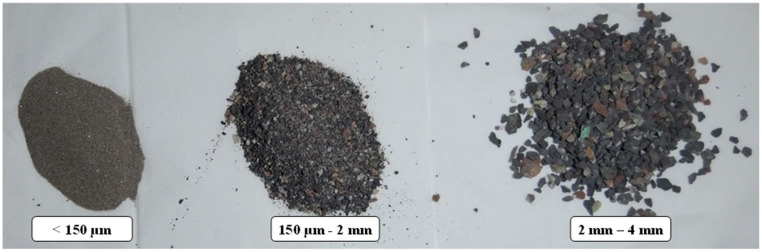
Wasted mineral aggregates in different sizes.

Mineral aggregates (quartz) in different diameters and with constant grain size are used in PC materials. The advantages of these wasted mineral aggregates include great bonding to polymers and high compressive strength. In Table 2, the chemical composition of this type of aggregate is presented. It should be noted that in order to remove the natural oil and wax that covers the surface of the mineral aggregates, before using them, their surface was treated and they were placed under a temperature of 100 °C for 3 h to dry. According to a previous study [36], aggregates with different weight percentages and sizes (including 50 wt% with 2–4 mm, 20 wt% with 150 µm–2 mm, and 5 wt% under 150 µm) were used to manufacture the samples. Figure 1 shows the different sizes of the aggregates used in this study. To increase the mechanical properties and improve the performance of PC materials, glass fibers and metal chips obtained from the machining process [37] were used. The metal chips used in this study were brass, and they were cleaned with acetone and cured at 50 °C for 30 min. Glass fiber was used from the composite sample that was made in advance. It is worth mentioning that if high-viscosity resin was utilized to manufacture the PC, the ingredients, particularly chopped glass fiber or aggregates, may be agglomerated. Therefore, in this study, low-viscosity (900–1100 cP at 25 °C) resin was used. In addition, in order to have a uniform mixture, all ingredients with their weight fractions were mixed inside a container using a mechanical stirrer for 5 min.

**Table 1:** Mechanical properties of EPL 1012 and EPH 112 [38].

Tensile Strength (kgf/cm^2^)	761
Tensile Modulus (kgf/cm^2^)	27,890
Flexural Strength (kgf/cm^2^)	960
Flexural Modulus (kgf/cm^2^)	36,454
Shore Hardness	82
Viscosity at 25 °C (mPa.s)–EPL 1012	900–1100
Viscosity at 25 °C (mPa.s)–EPH 112	30

### 2.2. Sample Geometry and Test Conditions

**Figure 2:**
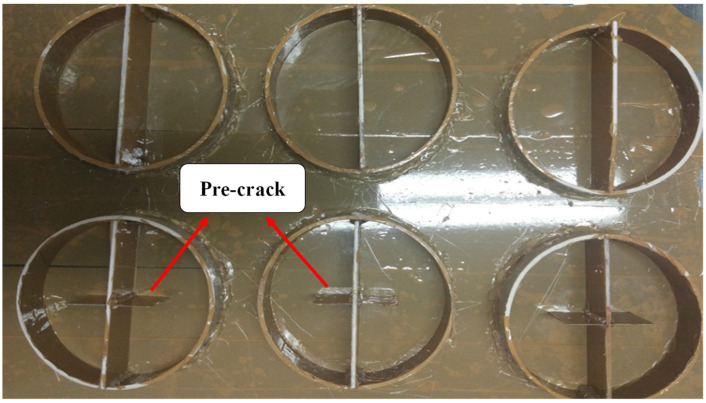
Circular molds used to make SCB specimens.

**Figure 3:**
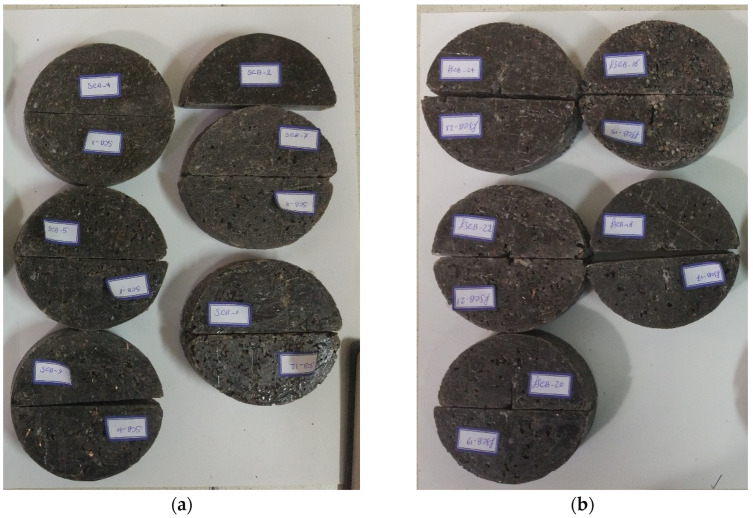
SCB samples with and without pre-crack for (**a**) strength and (**b**) fracture tests.

Researchers have used samples with different geometries for fracture and strength tests on PCs [39,40,41,42,43,44]. In this study, SCB samples were used to obtain the mode I fracture toughness, energy absorbed until failure, and tensile strength of PC materials. SCB samples were made from a mixture of epoxy resin, waste mineral aggregates, and fillers (brass chips and glass fibers). Figure 2 shows the circular molds used to manufacture the samples, which were created from PVC pipe cuts. Samples with a diameter of 105 mm and thicknesses of 30 mm and 22 mm were used for fracture and strength tests, respectively. An edge pre-crack was created by using an aluminum sheet with a thickness of 0.3 mm. The ratio of the crack length to the radius of the specimen was considered as 0.4. After one day, the specimens were taken out from the mold. The samples were then cured at 90 °C for 2 h. It should be noted that the samples were kept at room temperature for 7 days before the experiment. SCB samples with pre-crack were used for the fracture test and samples without cracks were used for the strength analysis. Figure 3 shows the test specimens. Table 3 also shows the details of the ingredients of the samples. For each condition, three samples were manufactured and tested.

### 2.3. Data Reduction Approach

**Figure 4:**
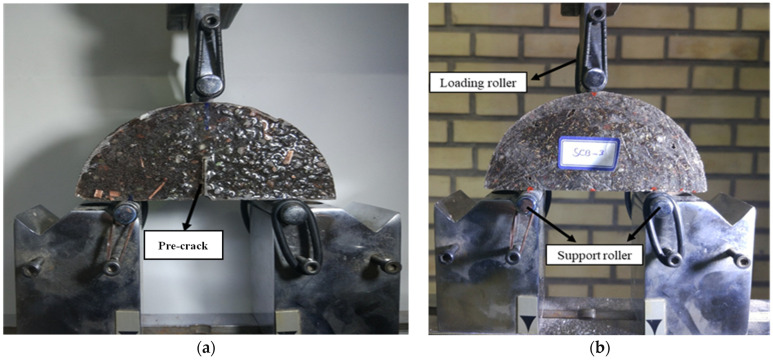
Test setup for (**a**) fracture test and (**b**) strength test (with no pre-crack).

An STM-150 universal test machine (Santam company, Tehran, Iran), with a capacity of 250 kN, was used for fracture and strength tests to obtain load–displacement curves of the specimens. It should be noted that the type of loading was displacement control and a speed of 0.5 mm/min was used [45]. The test setup and specimen under load are shown in Figure 4. The tensile strength was obtained from Equation (1) [11]:

(1) $$\sigma_{t}\left(\mathrm{SCB}\right)=\frac{P_{f}}{\pi tR}\left[0.073\;\left(\frac{t}{R}\right)-0.8896\right]\left[2.01\;\left(\frac{S}{R}\right)+1.052\right]$$

where *P_f_* and *t* are the maximum fracture load and thickness for the SCB specimen, respectively, *S* is the span length, and *R* is the radius of the specimens. To obtain the energy absorbed until failure, the area under the load–displacement curve of each test was calculated. Using Equation (2), the amount of absorbed energy can be extracted using the equation below:

(2) $$J_{f}=\int_{0}^{\Delta u}P.du$$

where *P* is the applied load, *J_f_* is the energy absorbed until failure, and *u* is the displacement measured by the testing machine. 

The fracture load in each of the samples was obtained by the test. Using Equation (3), the mode I fracture toughness value (*K_IC_*) can be determined [46]:

(3) $$K_{IC}=\frac{P_{C}\sqrt{a}}{RB\sqrt{\pi }}Y_{I}(a,R)$$

where *P_c_* is the fracture load, *R* is the disc radius, *B* is the sample thickness, *a* is half the length of the crack, and *Y_I_* is the geometric factor for mode I loading of SCB specimens, which is a function of the crack length-to-radius ratio. In this study, in order to determine the geometric factor for the tested setting, the figures reported by Ayatollahi and Aliha [46] were used. According to the mentioned explanations, by using the size of SCB samples (Section 2.2) and the fracture load obtained from the experimental tests, the fracture toughness of the samples was obtained. 

## 3. Results and Discussion

**Figure 5:**
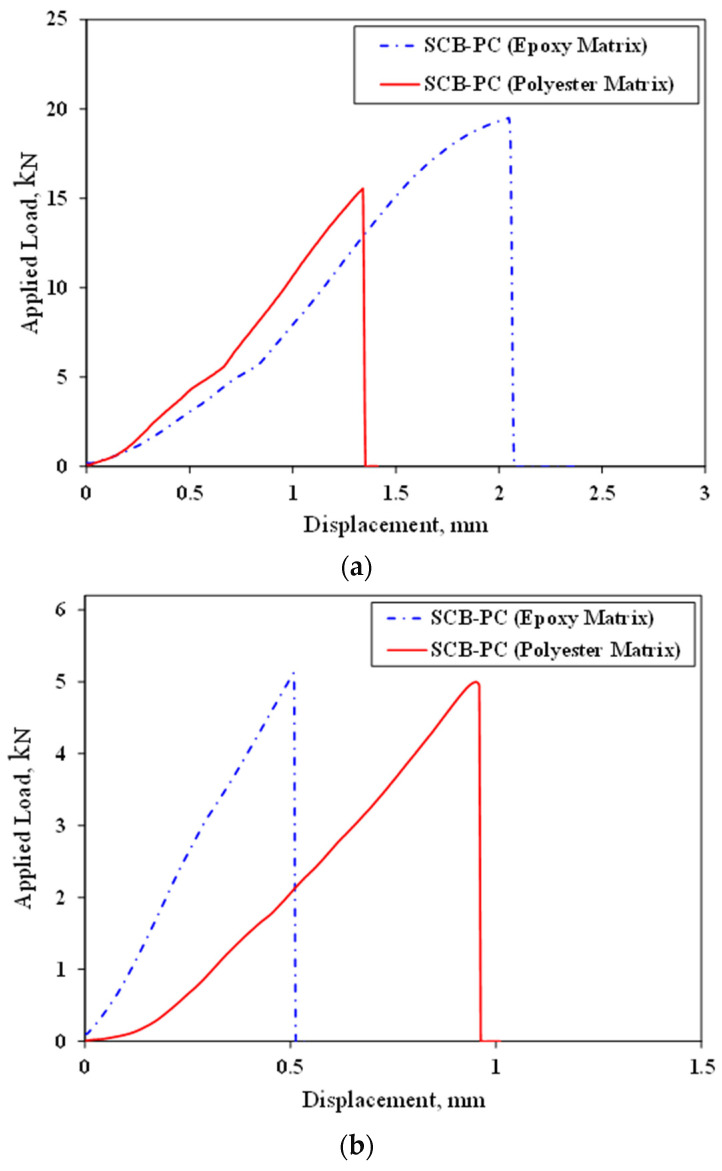
Typical load–displacement curves obtained for polymer concrete samples manufactured using two different resins: (**a**) strength and (**b**) fracture tests.

**Figure 6:**
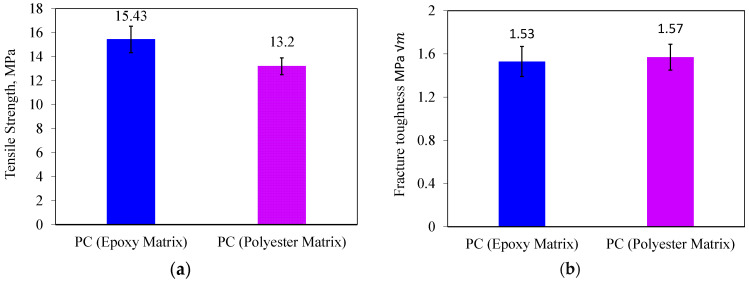
Tensile strength (**a**) and fracture toughness (**b**) of polymer concrete samples manufactured with two different matrices.

To select one of the resins considered in this study, SCB PCs without fillers were made using two matrices, epoxy and polyester. The tensile strength of neat epoxy and polyester are 74.6 MPa and 25.5 MPa, respectively [38,47]. Therefore, in the following, the PCs manufactured by two different matrices were analyzed in terms of strength and fracture toughness. In other words, the polymer concrete samples in this section have 75% mineral aggregates and 25% matrix; in one of the samples, epoxy was used as matrix, and in the other sample, polyester was used as matrix. Figure 5 shows typical load–displacement curves for each condition. In these curves, displacement was measured by the testing machine. Based on Figure 6, it is evident that the tensile strength of the PC made by the epoxy matrix is greater than that made by the polyester matrix. However, the fracture toughness values for both matrices are approximately equal. Additionally, it is important to mention that the epoxy resin has a longer gel time compared to the polyester matrix. The polyester matrix has a gel time of less than 10 min, which is unsuitable for fabricating PC specimens. Consequently, epoxy resin was chosen for the manufacture of the PC samples. 

### 3.1. Tensile Test Results

**Figure 7:**
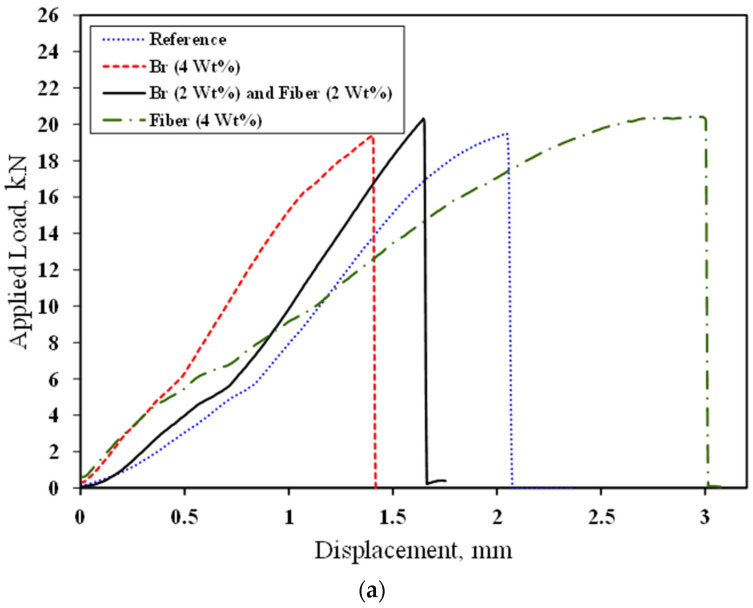

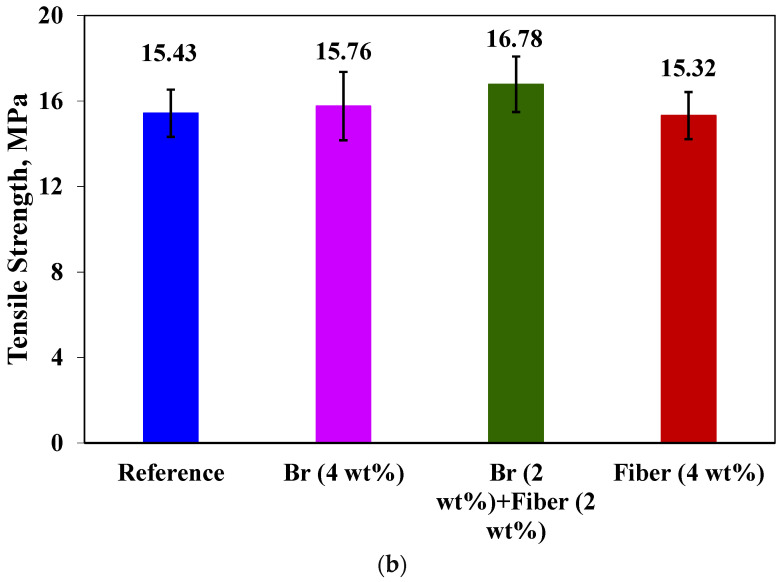
Load–displacement curves (**a**) and tensile strength (**b**) of SCB specimens.

**Figure 8:**
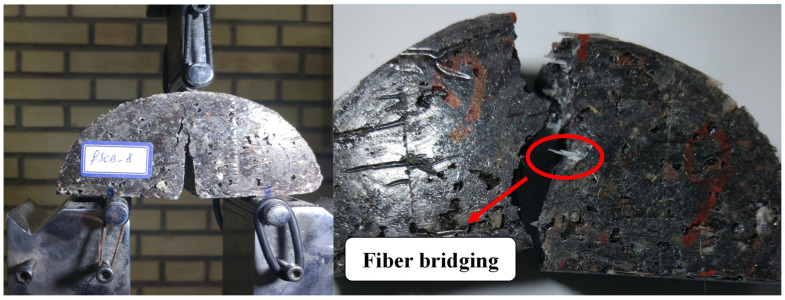
PC samples during the test and after fracture.

Figure 7 shows the typical load–displacement curves and tensile strength of PC with 4 wt% additives (different combinations). As shown, by adding brass chips and glass fibers, the tensile strength of the PC material does not change significantly. It should be noted that the reference legend in the following figures refers to PC manufacture by 75 wt% mineral aggregates and 25 wt% epoxy as matrix that does not contain any additives. Figure 8 shows the fracture surface of the tested samples. As crack growth is a gradual process, it does not break upon encountering the fibers. Instead, the matrix cracks elsewhere, resulting in a crack-arresting phenomenon.

### 3.2. Absorbed Energy

**Figure 9:**
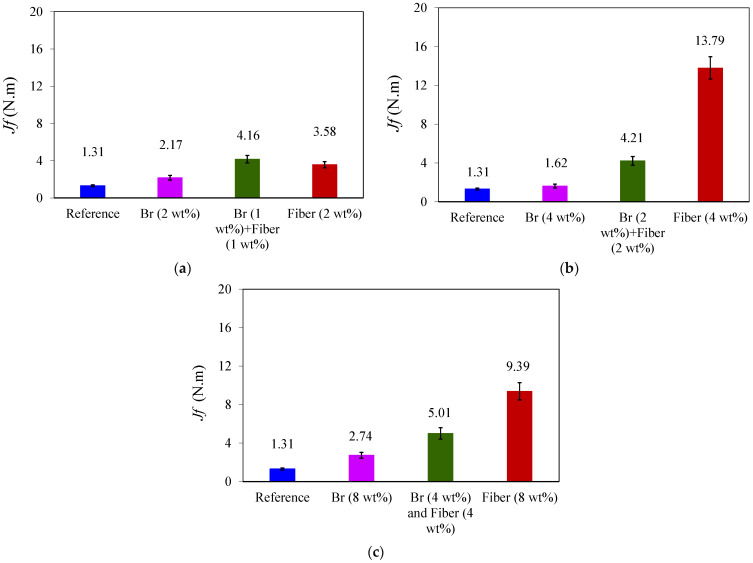
Average absorbed energy until the failure of SCB specimens for samples with (**a**) 2 wt%, (**b**) 4 wt%, and (**c**) 8 wt% additives.

Figure 9 shows the average energies obtained for samples with 2 wt%, 4 wt%, and 8 wt% additives. The highest absorbed energy until failure was obtained for PC with 4 wt% and 8 wt% glass fibers, as shown in Figure 9. The bridging mechanism observed in the specimens can be attributed to the increased energy absorption in the PC material. This phenomenon is a result of the addition of metal chips and glass fibers, which prevent rapid crack growth and resist it. Consequently, this factor contributes to an increase in absorbed energy.

### 3.3. Fracture Toughness

**Figure 10:**
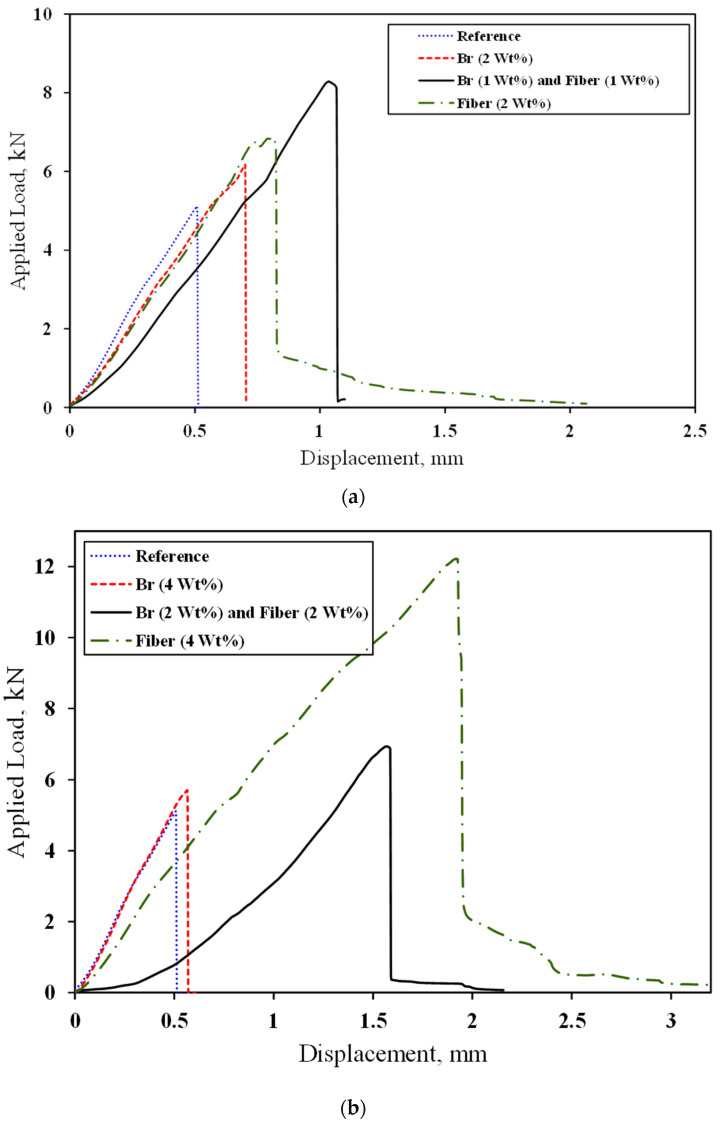

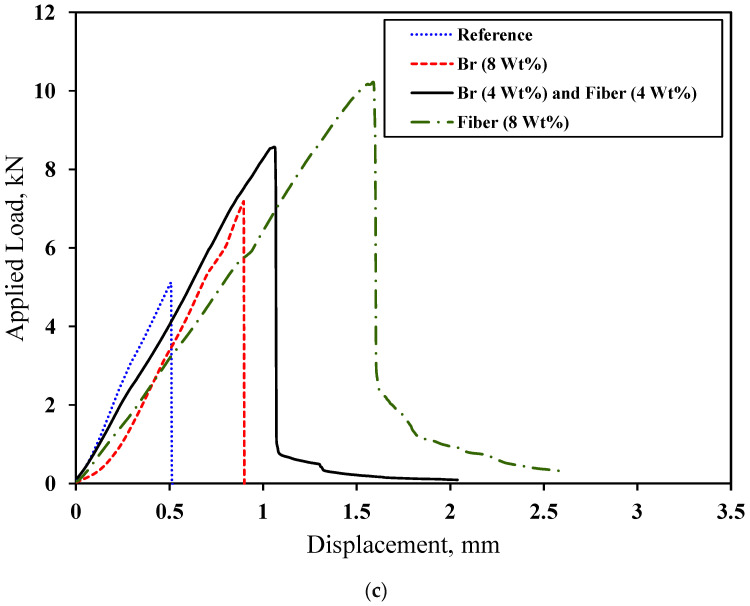
Load–displacement curves obtained for SCB samples with central crack in the states of (**a**) 2 wt%, (**b**) 4 wt%, and (**c**) 8 wt% under fracture test.

To assess the mode I fracture toughness, we conducted mode I fracture tests on SCB samples containing initial pre-cracks. It is important to highlight that in all the samples, failure originated from the crack tip. Figure 10 illustrates the load–displacement curves for samples with varying weight ratios of fillers, including 2 wt%, 4 wt%, and 8 wt%. Table 4 displays the maximum load and maximum displacement at failure in fracture tests. Notably, the sample containing 4 wt% fiber exhibited a significant improvement, with a 139% increase in maximum load and a remarkable 578% increase in displacement at the point of final failure. It is worth mentioning the gradual failure observed in the SCB samples, which effectively mitigates abrupt failure.

**Figure 11:**
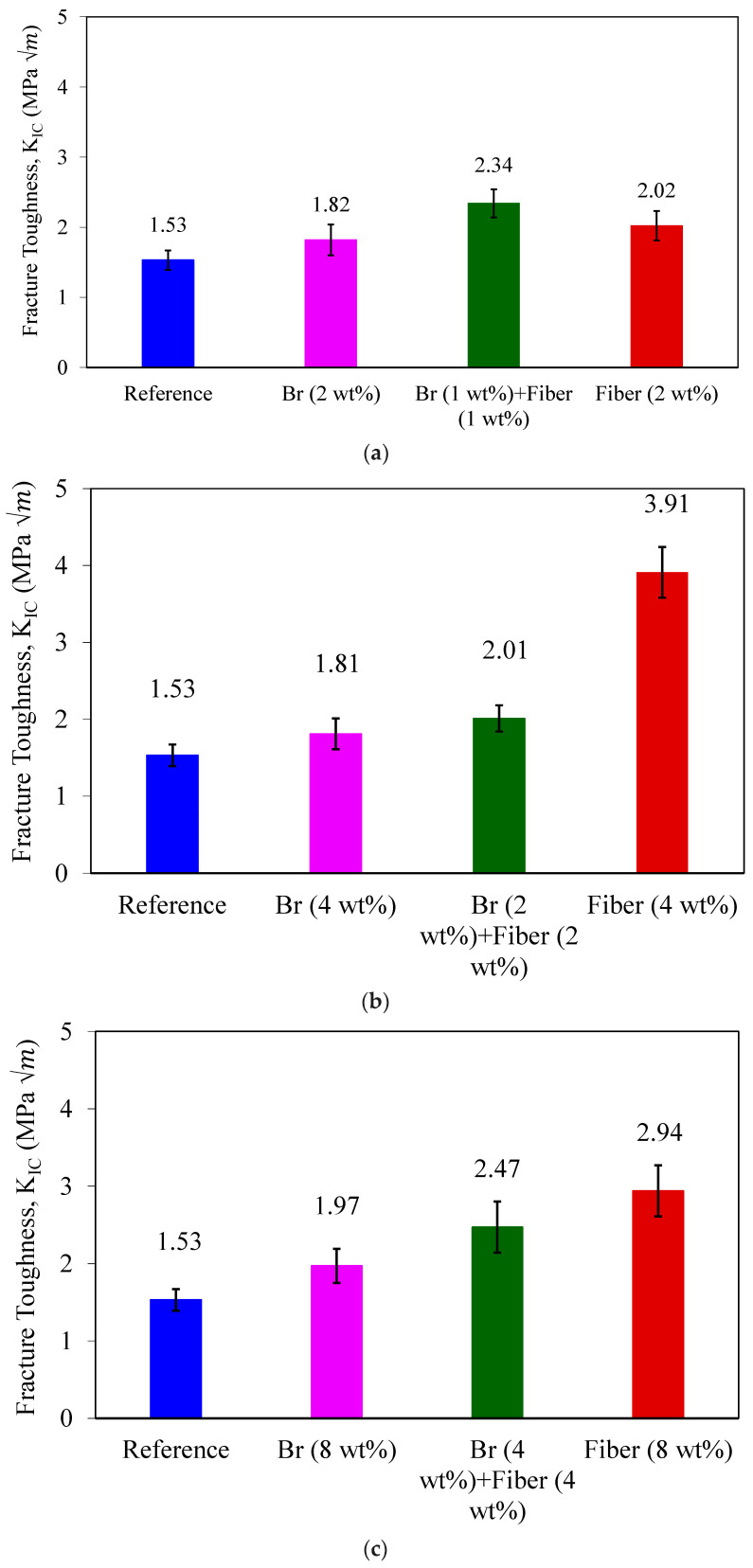
Average mode I fracture toughness of SCB specimens in the states of (**a**) 2 wt%, (**b**) 4 wt%, and (**c**) 8 wt% under fracture test.

In Figure 11, the average fracture toughness values, determined using Equation (3), are presented. According to the obtained results, the mode I fracture toughness increased by 32% with the addition of 2 wt% brass chips, while 1 wt% glass fibers + 1 wt% brass chips improved the fracture energy by 53%. Also, the addition of 4 wt% glass fibers increased the fracture toughness by 156%. Furthermore, the incorporation of 4 wt% glass fibers along with 4 wt% brass chips resulted in a remarkable 61% increase in fracture toughness. Nevertheless, the most exceptional performance was achieved when solely glass fibers were introduced to the PC samples, yielding an impressive 92% improvement in fracture energy. Based on the experimental findings, it can be deduced that the addition of glass fibers exerts the most significant influence on the fracture energy of the tested PCs. Notably, the introduction of 4 wt% glass fibers demonstrates the most substantial effect in enhancing the mode I fracture toughness of the tested concretes. 

**Figure 12:**
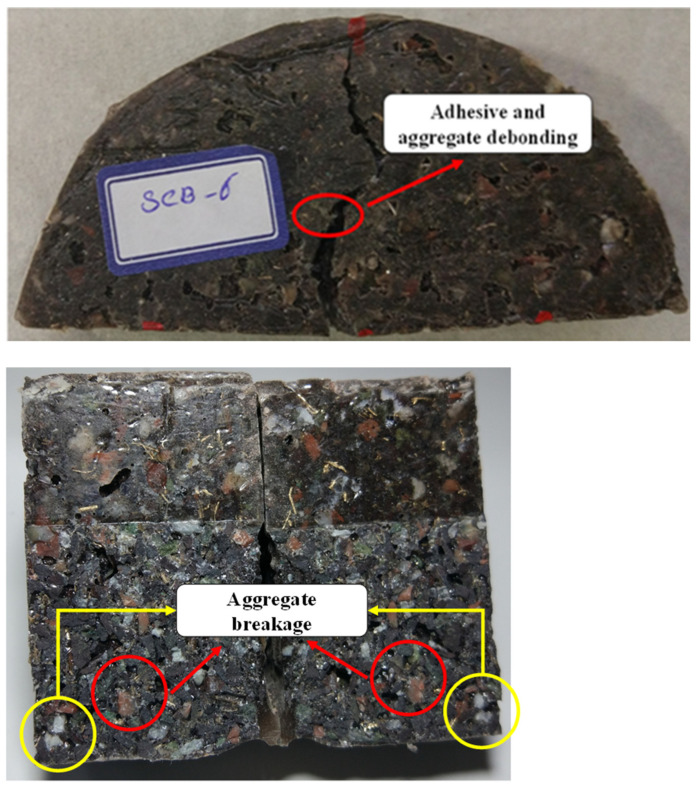
Fracture surface of SCB specimens with a central crack.

In Figure 12, the fracture surface of SCB specimens cracked under mode I loading is presented. The path of crack propagation observed in both strength and fracture tests originates from within the aggregates and at the interface between the aggregate and resin. The predominant failure mechanism identified in this study encompasses aggregate breakage, as well as adhesive and aggregate debonding. In fact, in samples without fillers, the predominant mechanism that inhibits crack growth or damage is aggregate fracture and debonding between the aggregate and adhesive. However, in samples containing fillers, in addition to these two mechanisms, the presence of fibers or metal chips can further impede crack growth and lead to multiple site cracking within the sample (see Figure 8).

## 4. Conclusions

In this study, the experimental investigation aimed to assess the influence of metal chips and glass fibers as fillers on the mode I fracture toughness, tensile strength, and energy absorption until failure of the PC material. SCB samples were fabricated using epoxy resin and waste mineral aggregates, with variations including 10 groups without filler and incorporating different weight ratios of various filler types, such as glass fibers, discarded metal chips, and various combinations of these materials. Quasi-static 3PB tests were conducted on samples without pre-cracks to determine the tensile strength of the SCB samples. The experimental results found that:

- The addition of fillers had minimal influence on tensile strength.
- The addition of 4 wt% glass fibers increased the energy absorption by 953%.
- Glass fibers have more impact on the mechanical response of the tested PCs compared to brass chips, and the inclusion of 8 wt% glass fibers substantially improved (by 92%) the resistance of the PC components to crack propagation.
- At lower weight ratios, a combination of brass chips and glass fibers could enhance the fracture response of the tested PCs.
- In unfilled samples, the main mechanism preventing crack propagation is aggregate fracture and the separation of the aggregate from the adhesive. Yet, in filled samples, alongside these mechanisms, the presence of fibers or metal chips adds an extra barrier to crack advancement, resulting in multiple-site cracking within the sample.

## Figures and Tables

**Table 2:** Chemical compounds of mineral aggregates [32].

Compounds	wt%
SiO_2_	97.5
Fe_2_O_3_	0.85
Al_2_O_3_	0.95
K_2_O	0.19
CaO	0.27
MgO	0.24

**Table 3:** Weight percentage of the components used in PC materials.

Sample Configuration	Mineral Aggregate (wt%)	Epoxy Resin (wt%)	Glass Fiber (wt%)	Brass Chip (wt%)
PC–No additive	75	25	0	0
Br (2 wt%)	73	25	0	2
Br (1 wt%) and Fiber (1 wt%)	73	25	1	1
Fiber (2 wt%)	73	25	2	0
Br (4 wt%)	72	24	0	4
Br (2 wt%) and Fiber (2 wt%)	72	24	2	2
Fiber (4 wt%)	72	24	4	0
Br (8 wt%)	70	22	0	8
Br (4 wt%) and Fiber (4 wt%)	70	22	4	4
Fiber (8 wt%)	70	22	8	0

**Table 4:** Maximum load and displacement at the final failure point for SCB samples.

Sample No.	Maximum Load, *P* (N)	Displacement at Final Failure, *δ* (mm)
Reference (PC with no additives)	5119	0.54
Br (2%)	6174	0.70
Br (1%) and Fiber (1%)	8289	1.07
Fiber (2%)	6865	2.06
Br (4%)	5706	0.60
Br (2%) and Fiber (2%)	6941	2.15
Fiber (4%)	12,222	3.65
Br (8%)	7183	0.90
Br (4%) and Fiber (4%)	8571	2.04
Fiber (8%)	10,224	3.089

## Data Availability

No new data were created or analyzed in this study. Data sharing is not applicable to this article.
